# Optimization of the Fermentation Media and Parameters for the Bio-control Potential of *Trichoderma longibrachiatum* T6 Against Nematodes

**DOI:** 10.3389/fmicb.2020.574601

**Published:** 2020-09-30

**Authors:** Shuwu Zhang, Yantai Gan, Jia Liu, Jingjiang Zhou, Bingliang Xu

**Affiliations:** ^1^Gansu Provincial Key Laboratory of Arid Land Crop Science, Gansu Agricultural University, Lanzhou, China; ^2^College of Plant Protection, Gansu Agricultural University, Lanzhou, China; ^3^Biocontrol Engineering Laboratory of Crop Diseases and Pests of Gansu Province, Lanzhou, China; ^4^Agriculture and Agri-Food Canada, Swift Current Research and Development Centre, Saskatchewan, SK, Canada

**Keywords:** *Trichoderma* species, *Heterodera avenae*, nematicidal activity, fermentation media and conditions, Plackett–Burman design, Box–Behnken design, response surface methodology

## Abstract

The cereal cyst nematode *Heterodera avenae* is one of the important soil-borne pathogens of cereal crops and causes high yield losses worldwide. *Trichoderma* spp. formulations are applied as commercial bio-control agents against soil-borne plant pathogens such as *H. avenae*. However, the relationship between *Trichoderma longibrachiatum* fermentation parameters and its bio-control potential against *H. avenae* has not been exclusively established. In the present study, the effect of 10 different fermentation media and conditions on the nematicidal activity of *T. longibrachiatum* T6 (T6) was evaluated with a single-factor method and a Plackett–Burman design, and the interaction between different fermentation parameters was investigated by a Box–Behnken design. The variables for enhancing the nematicidal activity of T6 culture filtrates were explored and optimized using response surface methodology (RSM). The Minor Medium (MM) plus wheat bran-2 medium was found to be the most effective fermentation medium for T6 culture filtrates against the second stage juveniles (J2s) of *H. avenae*. The maximum mortality of the J2s was obtained using the T6 culture filtrates under the following fermentation conditions: initial pH 6, 28°C culture temperature, 180 rpm rotating speed, 60 ml of fermentation media, 7 days of incubation time, and 1 ml of inoculation volumes. Among these parameters, the initial pH, inoculation volume, and incubation day were identified as the most significant parameters and critical independent variables for enhancing the nematicidal activity of the T6 culture filtrates. After further optimizations based on statistical predictions, the highest nematicidal activity (92.42%) was obtained with the T6 culture filtrates fermented under an initial pH of 6.06, an inoculation volume of 1.62 ml, and an incubation time of 7.15 days. The nematicidal activity was increased approximately by as high as 1.07% compared with that before optimization. Bio-control efficacy of T6 culture filtrates was 83.88% at the 70^th^ day after wheat seeds sowing in greenhouse experiments. The results from the validation experiments agreed with the model predictions. Our study has improved the bio-control potential of *Trichoderma* spp. against the plant-parasitic nematodes *H. avenae* and provided a cost-efficient bio-resource in the future development of novel bio-control agents.

## Introduction

The cereal cyst nematode (CCN, *Heterodera avenae*) is a plant pathogen found in cereal crops in more than 30 countries (Nicol et al., 2003; Jones et al., 2013; Baklawa et al., 2017). The pathogen causes significant crop yield losses (Long et al., 2012), particularly of rainfed cereals (Nicol and Rivoal, 2008). In China, *H. avenae* is distributed throughout nearly all cereal-growing areas including 13 provinces and autonomous regions (Peng et al., 2009; Niu et al., 2016), where the average yield loss due to *H. avenae* damage was estimated to range from 20 to 30% (Peng et al., 2009). Similarly, in Pakistan, Saudi Arabia, Australia, and United States, the crop yield losses due to CCNs were, respectively, 15–20, 40–90, 23–50, and 24% (Nicol et al., 2003; Smiley et al., 2005).

Strategies to manage plant parasitic nematodes in cropping systems have been mainly relied on chemical nematicides (Huang et al., 2014); such a method often causes concerns to environmental sustainability because of chemical pollution. Bio-management strategies have recently become a more preferred approach to reduce chemical hazards and to conserve the biodiversity of microbial communities (Sharma et al., 2014). Thus, the development of bio-control agents for plant diseases in agriculture has attracted more attention to minimize the use of chemical nematicides (Sergio, 2011). Some beneficial microorganisms, such as nematophagous bacterial and fungal species, are considered as ecologically friendly bio-control agents for the control of nematodes (Jatala, 1986). For example, nematode-trapping fungi, parasitic fungi, and pathogenic fungi are important parasites and predators as natural enemies against nematodes (Siddiqui and Mahmood, 1996; Kerry, 2001). The entomopathogenic fungi of *Paecilomyces* and *Pochonia* genus in nematode-suppressive soils have also been reported to have potential to combat plant parasitic nematodes (Nitao et al., 2002; Sharon et al., 2007; Mukhtar et al., 2013).

Recently, there has been an increasing research interest in applying microbial fermentations to manage plant parasitic nematodes worldwide (Khan et al., 2004; Yang et al., 2007). A growing number of studies have been devoted to analyze the potential of extracellular hydrolytic enzymes and secondary metabolites produced by some bio-control microorganisms to control target nematodes (Yang et al., 2016; Sharma et al., 2017). Studies have shown that *Trichoderma* spp. plays an important role in the development of bio-control agents because they are widely distributed, and mycotrophically filamentous and fast-growing fungi in soil (Harman et al., 2004). *Trichoderma* species, such as *T. harzianum*, *T. lignorum*, *T. koningii*, *T*. *virens*, and *T. viride*, have been applied as bio-control agents to suppress the populations of *Meloidogyne* spp. (Khan and Saxena, 1997; Spiegel and Chet, 1998; Sharon et al., 2001; Suarez and Llobell, 2004). It was shown that the culture filtrates of *T*. *virens* G1–3 could inhibit the egg hatching and cause second stage juveniles (J2s) mortality in *M. incognita* (Meyer et al., 2001). However, there is little information in the process of microbial fermentation regarding the components of fermentation medium and conditions used in obtaining these microorganisms and the relationship between the parameters in the process of microbial fermentation and the microorganisms’ ability against plant parasitic nematodes. Also, there is an urgent need of information on how to improve the bioactivity of the microorganisms against the nematodes.

Response surface methodology (RSM) has been reported as one of the preferred amenable approaches for determining the best culture conditions and the most effective components of the culture medium with a minimum number of experiments (Larentis et al., 2014; El-Naggar et al., 2016). In some cases, the RSM approach can help to prevent the possible misinterpretation of results when different variables interact with each other (Araujo and Brereton, 1996; Baş and Boyaci, 2007). In other cases, this widely used statistical technique can assist the enhancement of bioprocess optimization with the ability to circumvent the impediments associated with conventional methods (Cui et al., 2006).

In our previous work, we found that the strain of *T. longibrachiatum* T6 (T6) had a significant parasitic and lethal effect on the nematodes of *H. avenae* and *M. incognita* with a great potential to be used as a bio-control agent. However, the effectiveness of T6 culture filtrates against *H. avenae* J2s varies largely with fermentation media and conditions used (Zhang et al., 2014, 2015). There is a practical challenge in the development of the best filtrates and optimal fermentation conditions under which the nematicidal activity against *H. avenae* J2s can be assessed quantitatively.

The objectives of the present study, therefore, were to (i) maximize the nematicidal activity of T6 culture filtrates against *H. avenae* by evaluating the effect of different fermentation media and parameters on *H. avenae* mortality using the one-factor-at-a-time method and the Plackett–Burman design, and (ii) optimize the critical fermentation parameters to maximize the nematicidal activity of T6 culture filtrates using the RSM method. It is expected that the optimized assessment methodology will facilitate the development of commercial products for *H. avenae* bio-control.

## Materials and Methods

### Fungal Strain, Nematode Inoculum, and Media Preparation

The strain of *T. longibrachiatum* T6 (T6, CGMCC No.13183) was originally isolated from rhizosphere soil in Gansu, China (Zhang et al., 2017), and the spore suspension of the strain was prepared and stored in 20% glycerol at −80°C in the Laboratory of Plant Pathology, Gansu Agricultural University.

*H. avenae* cysts were isolated using the “Flotation separation” method (Long et al., 2012) from the soil samples collected from a wheat field in Xingyang, Henan province, China. Freshly hatched J2s were obtained following the procedure of Zhang et al. (2017). The suspension for inoculation was prepared at the concentration of 2 ± 1 J2s per 10 μl.

Ten different induction media (A, B, C, D, E, F, G, H, I, and J) (Table 1) were prepared and used to screen the optimum fermentation media. Each of these media (1 L) contains different compounds and is assumed to be beneficial for microorganisms against plant pathogens.

**TABLE 1:** Components of the fermentation media of *Trichoderma longibrachiatum* T6 used in the study.

Code	Media name	Media compounds	Total volume (ml)
A	Synthetic medium-1 (SM1)	[g/L]: (NH_4_)_2_SO_4_, 2.8 g; Urea, 0.6 g; KH_2_PO_4_, 4.0 g; CaCl_2_⋅2H_2_O, 0.6 g; MgSO_4_, 0.2 g; FeSO_4_⋅7H_2_O, 0.01 g; ZnSO_4_⋅H_2_O, 0.0028 g; CoCl_2_⋅6H_2_O, 0.0032 g; Glucose, 5.0 g	1,000
B	MM + wheat straw	[g/L]: Bran, 6 g; Inorganic salt solution, 4 g; 15 ml [% (w/v): KH_2_PO_4_, 1.25 g; (NH_4_)_2_SO_4_, 1.25 g; MgSO_4_, 0.3 g; CaCl_2_, 0.3 g]	1,000
C	Czapek’s Dox liquid medium (CDLM)	[% (w/v)]: Sucrose, 3%; NaNO_3_, 0.3%; KCl, 0.05%; MgSO_4_, 0.05%; KH_2_PO_4_, 0.1%; FeSO_4_, 0.001%	1,000
D	Synthetic medium-2 (SM2)	[g/L]: Fresh potato, 200 g; Mannitol, 40 g; Peptone, 6 g	1,000
E	MM + Wheat bran-1	[g/L]: Wheat bran, 7 g; Salt solution, 3 ml [% (w/v): NH_4_NO_3_, 0.5%; KH_2_PO_4_, 0.2%; NaCl, 0.1%; MgSO_4_⋅7H_2_O, 0.1%]	1,000
F	Potato dextrose broth (PDB)	[g/L]: Fresh potato, 200 g; Dextrose, 15 g	1,000
G	MM + Wheat bran-2	[g/L]: Wheat bran, 25 g; KH_2_PO_4_, 1 g; K_2_HPO_4_, 1.5 g; MgSO_4_⋅7H_2_O, 2 g; FeSO_4_⋅7H_2_O, 0.1 g	1,000
H	MM + Corn flour	[g/L]: Corn flour, 25 g; KH_2_PO_4_, 1 g; K_2_HPO_4_, 1.5 g; MgSO_4_⋅7H_2_O, 2 g; FeSO_4_⋅7H_2_O, 0.1 g	1,000
I	MM + Chitinase	NaCl, 10 mM; KCl, 10 mM; CaCl_2_, 3 mM; [% w/v]: 0.4% chitin	1,000
J	MM + Chitinase + Proteinase	NaCl, 10 mM; KCl, 10 mM; CaCl_2_, 3 mM; [% w/v]: Vitellin, 0.2%; Chitin, 0.2%; Chicken egg yolk, 0.7%; Asparagine, 0.2%; NaNO_3_, 0.2%; Glucose, 2%	1,000

### Preparation of *T. longibrachiatum* T6 Culture Filtrates

The strain of T6 was cultured initially on potato dextrose agar (PDA) in petri dishes at 25°C for 7 days under 16/8 h day/night conditions. The spore suspension was prepared following the procedures of Zhang et al. (2014) with minor modifications. The density of the spores was prepared to 1.0 × 10^6^ spores per milliliter and stored at 4°C. The fermentation was performed by inoculating 1 ml of the spore suspension in a 150 ml Erlenmeyer flask that contains 60 ml of 1 of 10 testing media and incubated on a rotary shaker at 180 rpm and 25°C for 5 days. The pH of the culture medium was adjusted to 6.0 by adding appropriate amounts of HCl or KOH prior to sterilization. Each medium was replicated six times. After 5 days of incubation, the culture broths from different replications were mixed and filtered through Whatman No.1 filter paper. The culture filtrates were used as stock solutions for determining the effect of culture media on the nematicidal activity of T6 strain.

### The Nematicidal Activity Assay of T6 Culture Filtrates

*H. avenae* J2s and the stock solutions of T6 culture filtrates were used to assay the nematicidal activity *in vitro* against the J2s of *H. avenae*. Ninety milliliters of the stock solutions were mixed with two to three J2s in each well of a 96-well sterilized cell culture plate. The wells containing sterile water without T6 culture filtrates were used as the control. All the plates were then incubated at 25°C under 16/8 h day/night condition. The inactive J2s were allowed to recover in tap water for 5 h (Meyer et al., 2004) and then prodded with a needle, and those that did not respond were considered dead. The number of live and inactive nematodes was counted at 24, 48, and 72 h after the treatments. Each treatment including the control was replicated six times. The mortality of *H. avenae* J2s was calculated as follows (Zhang et al., 2015):

(1) M(%)=NDSSJ/TNSSJ×100

where M represents mortality, NDSSJ represents the number of dead J2s, and TNSSJ represents the total number of J2s used in each replicate of each treatment.

### Optimization of Fermentation Parameters by Single-Factor Experiments

To investigate the optimum fermentation parameters of T6 culture filtrates for the best nematicidal activity, six key fermentation parameters were studied individually by the quasi-optimum protocol (single-variable approach) to evaluate the effect of each fermentation conditions on the nematicidal activity of T6 culture filtrates, including (i) pH, (ii) incubation temperature, (iii) inoculation volume, (iv) liquid media volume, (v) rotating speed, and (vi) incubation day. Two controls were set: the J2s were treated with sterilized water as negative control, and the J2s were treated with the fermentation media without T6 strain as positive control.

The parameters were initially set as follows: pH 6.0; incubation temperature, 25°C; inoculation volume, 1 ml; incubation time, 5 days; fermentation volume, 60 ml; and rotating speed, 150 rpm. Then, the six parameters of the fermentation media were varied individually from their initial level. Each treatment was replicated six times, and the mean percentages of J2s mortality were calculated to assay the nematicidal activity of T6 culture filtrates. The fermentation media pH was adjusted to 4.0, 5.0, 6.0, 7.0, 8.0, and 9.0 with appropriate amounts of HCl or KOH and agar prior to sterilization. The incubation temperature was adjusted to 20, 25, 28, 30, and 35°C. The volume of inoculation was set at 0.5, 1.0, 1.5, 2.0, 2.5, and 3.0 ml in a 150 ml flask. The liquid media volume was set at 30, 40, 50, 60, 70, and 80 ml. The rotating speed was set at 100, 120, 140, 160, 180, and 200 rpm. The incubation day was adjusted from 4 to 9 days with a 1-day internal. The nematicidal activity of T6 strain culture filtrates was assessed by extracting samples from each fermentation condition.

### Screening Significant Fermentation Parameters by the Plackett–Burman Design

The Plackett–Burman design was used to screen the fermentation parameters that significantly influence the nematicidal activity of T6 culture filtrates under different fermentation conditions. The independent parameters used in the Plackett–Burman design were (i) pH (4.0 and 9.0), (ii) incubation temperature (20°C and 35°C), (iii) inoculation volume (0.5 ml and 3 ml), (iv) liquid media volume (30 ml and 80 ml), (v) rotating speed (100 rpm and 200 rpm), and (vi) incubation day (4 days and 9 days). They were experimentally screened with 12 trials at two levels: -1 for low level and + 1 for high level. The actual high and low values of each parameter with whole randomized experimental design are presented in Table 2. Each trail was conducted with six replicates and the mean mortality at 72 h was taken as the response. Minitab 17 statistical software (Minitab Inc., United States) was used as a tool for screening in the Plackett–Burman design. The first-order polynomial model was adopted to evaluate the effect of each independent variable to the response:

(2) $$Y=\beta_{0}+\Sigma \beta_{i}\chi_{i}$$

**TABLE 2:** PBD matrix for screening of independent variables with actual values affecting the nematicidal activity (mortality) of *Trichoderma longibrachiatum* T6 culture filtrates.

Run number	Initial pH	Incubation temperature (°C)	Inoculation volume (ml)	Liquid volume (ml)	Rotating speed (rpm)	Incubation day (days)	*Y*: Mortality (%)	
							Actual	Predicted
1	4	35	3	80	100	9	72.57	72.53
2	9	35	3	30	200	9	69.02	69.14
3	4	35	3	30	200	4	70.41	70.15
4	9	35	0.5	80	100	4	69.32	69.56
5	4	35	0.5	30	100	9	73.65	73.82
6	4	20	0.5	30	100	4	73.08	72.43
7	9	35	0.5	80	200	4	70.43	70.19
8	4	20	0.5	80	200	9	75.22	76.08
9	9	20	0.5	30	200	9	72.45	72.06
10	9	20	3	30	100	4	66.12	67.12
11	9	20	3	80	100	9	70.87	70.14
12	4	20	3	80	200	4	71.87	71.78

where *Y* is the response of mortality (%), β_0_ is the model intercept, β_*i*_ is the linear coefficient, and χ_*i*_ is the level of the independent fermentation parameters (*i* = i, ii, iii, iv, v, and vi).

### Optimizing Significant Fermentation Parameters by RSM

From the data sets obtained from the above Plackett–Burman design experiments, the Box–Behnken method (Design Expert software, version 10.7, Stat-Ease Inc., United States) was performed to identify fermentation parameters that have a significant effect on the bio-control potential of T6 culture filtrates and to evaluate the optimum level and interactive effects of the significant parameters influencing the nematicidal activity of T6 culture filtrates. These significant parameters were considered as the significant independent variables and used in the RSM to further optimize each of these significant parameters with two levels, high and low, denoted by (+1) and (−1), respectively, for the enhanced nematicidal activity of T6 culture filtrates (Table 3). The proper levels of each variable were determined based on the method for the single-factor experiment for the mortality of the J2s caused by T6 culture filtrates. A polynomial quadratic equation was adopted to evaluate the effect of each independent variable (*i* and *j*) to the response:

(3) Y=β0+Σβiχ+iΣβiiχi2+Σβijχiχj

**TABLE 3:** Values of independent variables and the levels used in central composite design for optimization of expression.

Code	Factors	Levels	
		−1	1
A	Initial pH value	4	9
B	Inoculation volume (ml)	0.5	3
C	Incubation day (days)	4	9

where *Y* is the measured response associated with each parameter by level combination: *β*_0_ is an intercept; *β*_*i*_ is regression coefficients computed from the observed experimental values of *Y*; and *X*_*i*_ is the coded levels of independent variables. The terms *X_*i*_X_*j*_* and *X*^2^_*i*_ represent the interaction and quadratic terms, respectively. *β*_*ij*_ is the quadratic coefficient and *β*_*j*_ is the *ij* interaction coefficient.

The predicted mortalities were verified experimentally as in “Preparation of *T. longibrachiatum* T6 culture filtrates” and “The nematicidal activity assay of T6 culture filtrates” with 12 replicates under each parameter combination. Statistical significance of the obtained equation was assessed by an *F*-test and the analysis of variance (ANOVA) for response surface quadratic model.

### Greenhouse Bio-control Experiment

The *H. avenae* susceptible wheat cultivar of Yongliang 4 was used to identify the effectiveness of T6 culture filtrates to control *H. avenae* in greenhouse experiment. The wheat seeds were sterilized and planted according to the method described by Zhang et al. (2017). Each pot had 10 seedlings, and they were irrigated with sterile distilled water every 2 days after seedling emergence. The experiment was arranged using a completely randomized design. When the seedlings reached 8 cm in height, each pot was inoculated with 1600 ± 100 J2s of *H. avenae*. Ten days after the inoculation, the seedlings were inoculated with 10 ml of T6 culture filtrates. The wheat seedlings inoculated with the same number of J2s and 10 ml of sterilized distilled water but without T6 culture filtrates were considered as the negative control, and seedlings inoculated with J2s and 10 ml of liquid fermentation medium were used as positive control. The wheat seedlings inoculated with the same number of J2s and 2 ml of 1.8% abamectin emulsifiable concentrate (EC) (2000 dilutions) were also included to compare with the T6 culture filtrate treatment. The inoculated seedlings were grown with supplemental day/night lighting of 16/8 h at 25°C, and relative humidity of 65%. Seventy days after seedling inoculation, the parameters of number of juveniles in soil and bio-control efficacy were recorded and determined.

### Statistical Analysis

Minitab Statistical Software (version 17, Minitab Inc., United States) was used to screen the significant parameters affecting the nematicidal activity. The response surfaces of the variables inside the experimental domain were analyzed using Design Expert software (Version 10.7, Stat-Ease Inc., United States). The quality of the fit to the polynomial model equation was expressed by the coefficient of determination *R*^2^, and the significances of the regression coefficient were checked by *F*-test and *p*-value. The values of “Prob > *F*” less than 0.05 indicate the significant model terms, while values greater than 0.10 indicate that the model terms are not significant. A large number of insignificant model terms could be improved by model reduction. The adequacy of the regression model was estimated by drawing the diagnostic plots. The *R*^2^ coefficient was calculated as the indicator of the model fitting. The model would be stronger and the prediction of the response would be better if the *R*^2^ coefficient approaches 1.

Data from experiments were subjected to ANOVA and expressed as means of standard errors (SE) of 6 or 12 replicates. One-way ANOVA was performed to determine the treatment effect using SPSS Version 16.0 (SPSS Inc., Chicago, IL). Treatment effects were determined using Duncan’s multiple range test and the significances were expressed at *p* < 0.05.

## Results

### Effect of Fermentation Media on the Nematicidal Activity of T6 Culture Filtrates

Compared with the control, the T6 cultural filtrates from 10 different fermentation media showed different degrees of nematicidal activity against *H. avenae* J2s. Among the fermentation media (Table 1), the T6 cultural filtrates from the MM + wheat bran-2 (G), MM + chitinase medium (I), and MM + chitinase + proteinase (J) had a higher nematicidal activity with mortalities of 91.44, 89.04, and 90.38%, respectively, after 72 h incubation. The T6 cultural filtrates from other four media, synthetic medium-1 (A), Czapek’s Dox liquid medium (C), MM + wheat bran-1 (E), and potato dextrose broth (F), produced mortalities from 80.06 to 88.75% after 72 h incubation. The T6 cultural filtrates from the MM + corn flour medium (H) exhibited the lowest mortality of only 62.94% after 72 h incubation. Thus, the MM + wheat bran-2 (G) was considered as the most effective fermentation medium for the T6 strain against *H. avenae* J2s (Table 4).

**TABLE 4:** Nematicidal activity (mortality) of *Trichoderma longibrachiatum* T6 culture filtrates against *H. avenae* with 10 different fermentation media.

Medium code	Time (h)		
	24	48	72

	Mortality (%)		
A	24.79 ± 1.06 e	54.33 ± 1.84 e	80.57 ± 2.56 d
B	27.10 ± 1.86 d	56.78 ± 2.33 de	73.55 ± 2.78 f
C	33.69 ± 2.67 bc	64.22 ± 3.07 b	80.06 ± 2.88 d
D	31.00 ± 1.98 c	63.39 ± 1.78 b	79.33 ± 2.34 e
E	34.14 ± 2.54 b	58.64 ± 3.03 d	88.75 ± 3.32 b
F	36.27 ± 1.46 a	60.34 ± 2.36 c	86.26 ± 2.88 c
G	31.37 ± 2.54 c	68.58 ± 3.26 a	91.44 ± 3.32 a
H	25.80 ± 1.22 e	44.18 ± 2.05 f	62.94 ± 3.20 g
I	28.00 ± 1.97 d	57.98 ± 2.38 d	89.04 ± 3.10 ab
J	34.29 ± 2.05 b	65.71 ± 3.07 b	90.38 ± 2.88 a
Control	7.46 ± 0.78 f	9.37 ± 0.78 g	10.67 ± 1.04 h

*Data are means ± standard error of six replicates and those in a column followed by different letters are significantly different at p < 0.05, based on Duncan’s new multiple range test using one-way ANOVA (n = 6). The mortality (%) was determined at 24, 48, and 72 h after inoculation of the J2s with either T6 fermentation or sterile water (control). The medium code is detailed in the footnote of Table 1.*

### Key Fermentation Parameters for Maximizing the Nematicidal Activity of T6 Culture Filtrates

**FIGURE 1:**
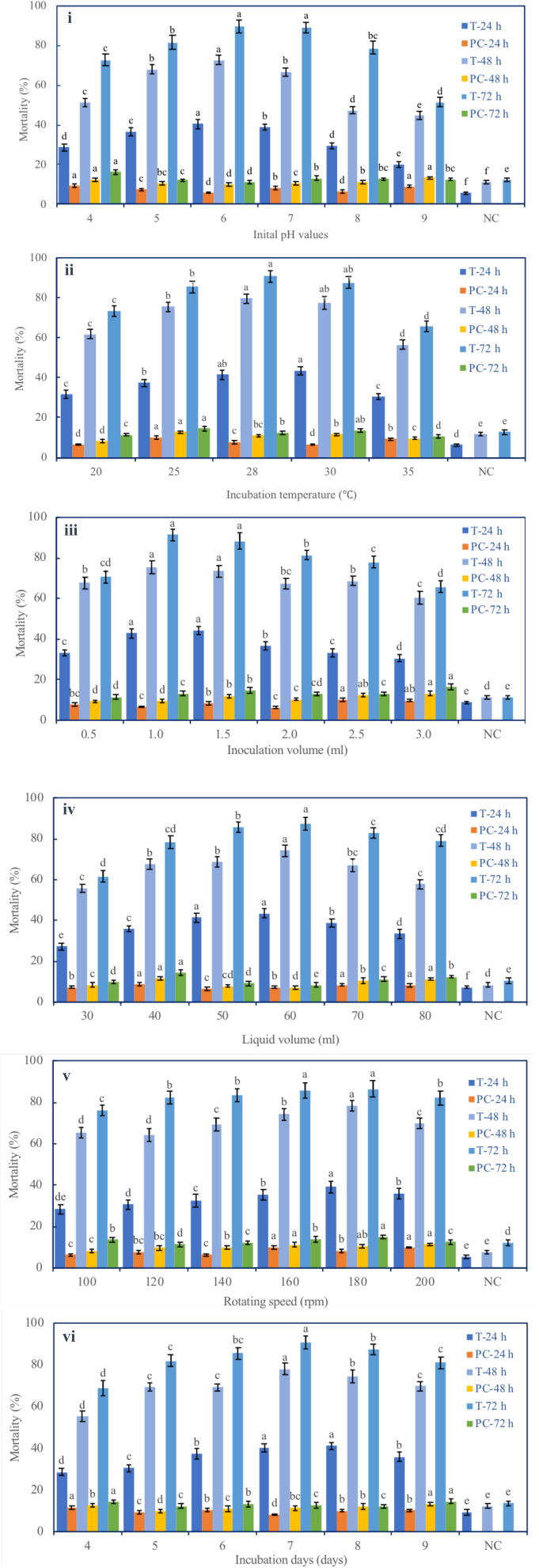
Effect of different fermentation parameters on the nematicidal activity of *Trichoderma longibrachiatum* T6 culture filtrates against *H. avenae.* The mortalities are shown under the parameters of **(i)** initial pH, **(ii)** incubation temperature, **(iii)** inoculation volume, **(iv)** liquid volume, **(v)** rotating speed, and **(vi)** incubation day. T represents T6 culture filtrate treatment. PC represents the J2s that were treated with the liquid fermentation medium but not with *T. longibrachiatum* T6 (positive control). NC represents the J2s that were treated with sterilized water (negative control). The keys are T-24 h, T-48 h, and T-72 h for T6 culture filtrate treatments, and PC-24 h, PC-48 h, and PC-72 h for the positive controls at 24 h, 48 h, and 72 h after the treatments, respectively. The data are grouped by the parameter, and there are six groups (i–vi). The statistical analysis was applied in each group on the mortality for each treatment (T-24 h, PC-24 h, T-48 h, PC-48 h, T-72 h, and PC-72 h). Data are means of six replicates ± standard error. The line bars represent the standard errors of the means. Different letters denote significant difference at *p* < 0.05 based on Duncan’s new multiple range tests using one-way ANOVA for a range of each fermentation parameter.

Six fermentation parameters (initial pH, incubation temperature, inoculation volume, liquid media volume, rotating speed, and incubation day) of the G medium (MM + wheat bran-2) exhibited a significant effect on the nematicidal activity of T6 culture filtrates (Figure 1). Increasing incubation time from 24 to 72 h significantly and continuously increased the nematicidal activity. After 72 h incubation with the G medium, the highest nematicidal activity was achieved: 89.67% at initial pH 6 (Figure 1i), 90.67% at fermentation temperature 28°C (Figure 1ii), 91.57% with inoculation volume 1 ml (Figure 1iii), 87.35% with liquid media volume 60 ml (Figure 1iv), 86.54% with rotating speed 180 rpm (Figure 1v), and 90.00% with incubation time 7 days (Figure 1vi).

### Contribution of the Fermentation Parameters in the Nematicidal Activity of T6 Culture Filtrates

**FIGURE 2:**
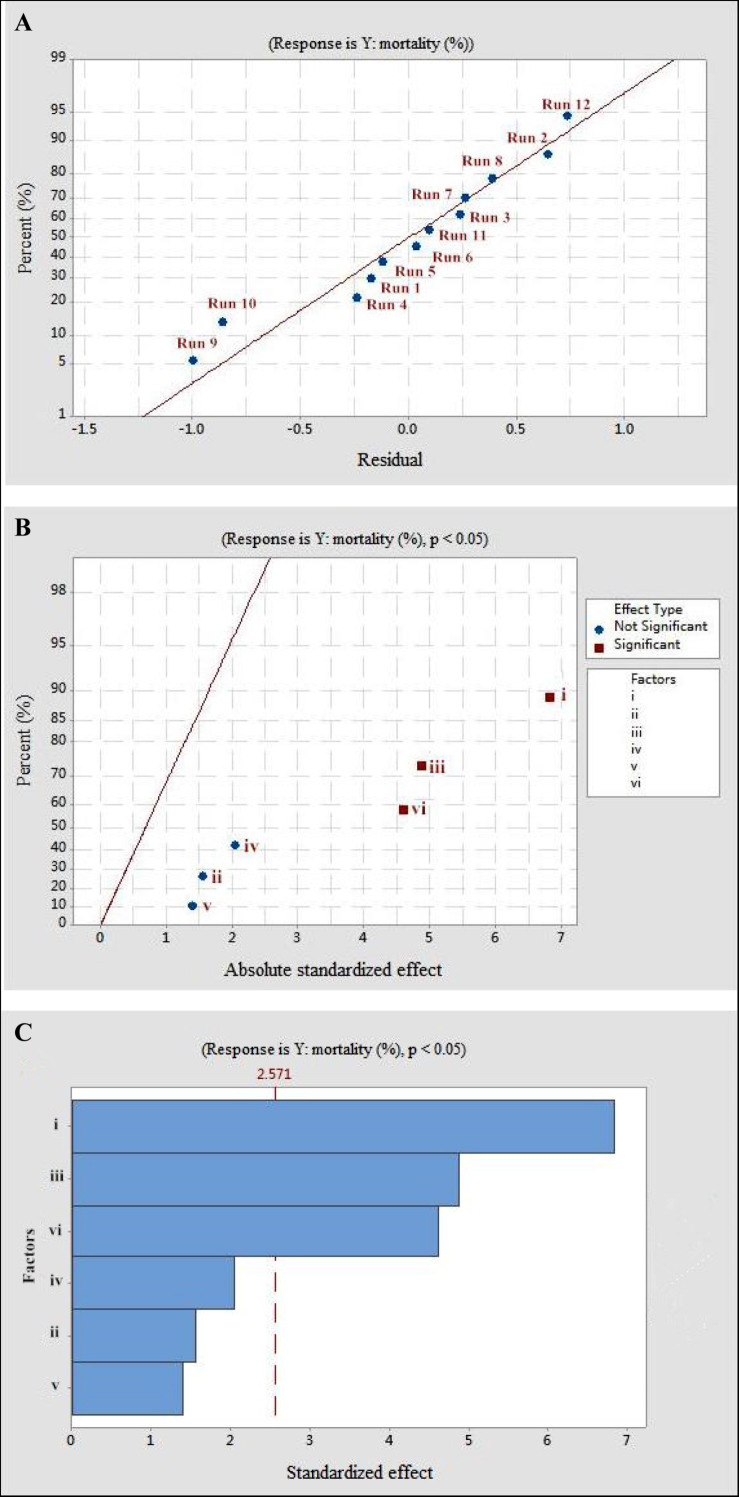
The mortality response to the effects of residuals, absolute standardized effect, and standardized effect on the nematicidal activity of *Trichoderma longibrachiatum* T6 against *H. avenae* as presented with **(A)** normal plot, **(B)** half normal plot, and **(C)** pareto chart presentation, with (i) initial pH, (ii) incubation temperature, (iii) inoculation volume, (iv) liquid volume, (v) rotating speed, and (vi) incubation day.

The Plackett–Burman design was used to screen the fermentation parameters (used as factors in the design) for their significant contribution to the nematicidal activity of T6 culture filtrates. In Plackett–Burman design experiments, the nematicidal activity (mortality) varied from 66.12 to 75.22%. The mortality of different parameter combinations after 72 h incubation under the influence of the parameters at high and low levels is shown in Table 2. The highest mortality (75.22%) was observed in the 12^th^ run with a combination of initial pH 4.0, fermentation temperature, 20°C; inoculation volume, 0.5 ml; incubation day, 9 days; rotating speed, 200 rpm; and inoculation volume, 80 ml. A high correlation was observed between these predicted mortalities and the experimental mortalities obtained in Table 2 (Figure 2A).

Statistical significance was assessed by an *F*-test and the ANOVA for Plackett–Burman design quadratic model. The first-order model was obtained with experimental results as follows: *Y* = 73.43 - 0.6197i - 0.0468ii - 0.886iii + 0.0185iv + 0.00632v + 0.4183vi, where *Y* is predicted response and i, ii, iii, iv, v, and vi are the coded values of initial pH, fermentation temperature, inoculation volume, liquid volume, rotating speed, and incubation day, respectively. The regression analysis indicated a significance of the model terms at *p* < 0.01 and a high correlation between the observed and predicted values between the independent parameters and the response (Table 5). The coefficient of determined *R*^2^ and adjusted *R*^2^ were 0.95 and 0.90, respectively. The initial pH, inoculation volume, and incubation day were predicted to have a significant and substantial influence on the nematicidal activity of T6 culture filtrates among all six fermentation parameters (Figures 2B,C and Table 5).

**TABLE 5:** Regression coefficient and analysis of variance for the quadratic model for the nematicidal activity (mortality) of *Trichoderma longibrachiatum* T6 culture filtrates.

Term	Effect	Coefficient	Df	Adj SS	Adj MS	*F*-value	*p*-value
Model			6	61.884	10.314	16.690	0.004*
Linear			6	61.884	10.314	16.690	0.004*
Constant		71.251					0.000*
i. Initial pH	–3.098	–1.549	1	28.799	28.799	46.600	0.001*
ii. Incubation temperature (°C)	–0.702	–0.351	1	1.477	1.477	2.390	0.183
iii. Inoculation volume (ml)	–2.215	–1.108	1	14.719	14.719	23.820	0.005*
iv. Liquid volume (ml)	0.925	0.462	1	2.567	2.567	4.150	0.097
v. Rotating speed (rpm)	0.632	0.316	1	1.197	1.197	1.940	0.223
vi. Incubation day (days)	2.092	1.046	1	13.125	13.125	21.240	0.006*
Residual error			5	3.09	0.618		
Total			11	64.974			

F is Fisher’s function; probability *p < 0.05 corresponds to significance.

### RSM Analysis of the Significant Parameters

Based on the single-factor experiments and the Plackett–Burman design experiments above, the initial pH, inoculation volume, and incubation day were selected as the independent variables in the response surface optimization (RSM) at two levels (−1 and + 1) (Table 3).

The optimization of the selected parameters was carried out through 17 experiments with five replications of the central points each experiment (Table 6). The quadratic polynomial model obtained by regression was: *Y* = 19.33A + 18.41B + 12.34C − 5.04AB + 4.00AC − 0.35BC − 1.60A^2^ − 4.91B^2^ − 0.82C^2^ − 25.46, which was used to calculate the predicted mortality as a function of initial pH value (A), inoculation volume (B), and incubation day (C).

**TABLE 6:** Box–Behnken design with the measured and predicted response of mortality in relation to the three key parameters of pH (A), inoculation volume (B), and incubation day (C).

Run number	Parameters			Y: Mortality (%)	
	A	B	C	Actual	Predicted
1	4.00	1.75	9.00	81.74	82.29
2	4.00	3.00	6.50	76.44	75.89
3	6.50	1.75	6.50	92.33	91.34
4	6.50	1.75	6.50	92.62	91.34
5	9.00	1.75	9.00	74.67	75.22
6	9.00	3.00	6.50	69.37	68.82
7	6.50	1.75	6.50	90.67	91.34
8	6.50	3.00	9.00	78.63	78.63
9	6.50	1.75	6.50	89.53	91.34
10	9.00	1.75	4.00	70.68	70.13
11	6.50	1.75	6.50	91.53	91.34
12	6.50	0.50	4.00	76.20	76.20
13	4.00	0.50	6.50	78.00	78.55
14	6.50	3.00	4.00	74.64	75.73
15	6.50	0.50	9.00	84.56	83.47
16	9.00	0.50	6.50	70.93	71.47
17	4.00	1.75	4.00	77.76	77.21

In order to understand the effect of the independent variables on the dependent variable, the response surface plots of the quadratic polynomial model were generated by varying one of the independent variables within the experimental range while holding other variables constant at the central point. The results of statistical assessment [*F*-value: 71.93 and *P* (Prob > *F*) < 0.0001] confirmed the significance of the model terms with regression coefficient constants *R*^2^ of 0.99 and Pred *R*^2^ of 0.92, which are in reasonable agreement with the Adj *R*^2^ of 0.98. The model terms A, B, C, A^2^, B^2^, and C^2^ could be considered as significant terms (Table 7). The RSM results showed that initial pH value (A), inoculation volume (B), and incubation day (C) could adequately describe the response (mortality).

**TABLE 7:** Analysis of variance (ANOVA) for response surface quadratic model for the nematicidal activity of *Trichoderma longibrachiatum* T6 culture filtrates.

Source	Sum of squares	df	Mean square	*F*-value	*p*-value
Model	1032.59	9	114.73	71.93	< 0.0001*
A. pH	100.04	1	100.04	62.72	< 0.0001*
B. Inoculation volume	14.07	1	14.07	8.82	0.0208*
C. Incubation day	51.61	1	51.61	32.36	0.0007*
AB	0.000	1	0.000	0.000	1.0000
AC	2.500E-005	1	2.500E-005	1.567E-005	0.9970
BC	4.77	1	4.77	2.99	0.1272
A^2^	418.78	1	418.78	262.57	< 0.0001*
B^2^	248.22	1	248.22	155.63	< 0.0001*
C^2^	111.70	1	111.70	70.03	< 0.0001*
Residual	11.16	7	1.59		
Lack of fit	4.79	3	1.60	1.00	0.4789
Pure error	6.38	4	1.59		
Cor total	1043.76	16			

F is Fisher’s function; probability *p < 0.05 corresponds to significance.

**FIGURE 3:**
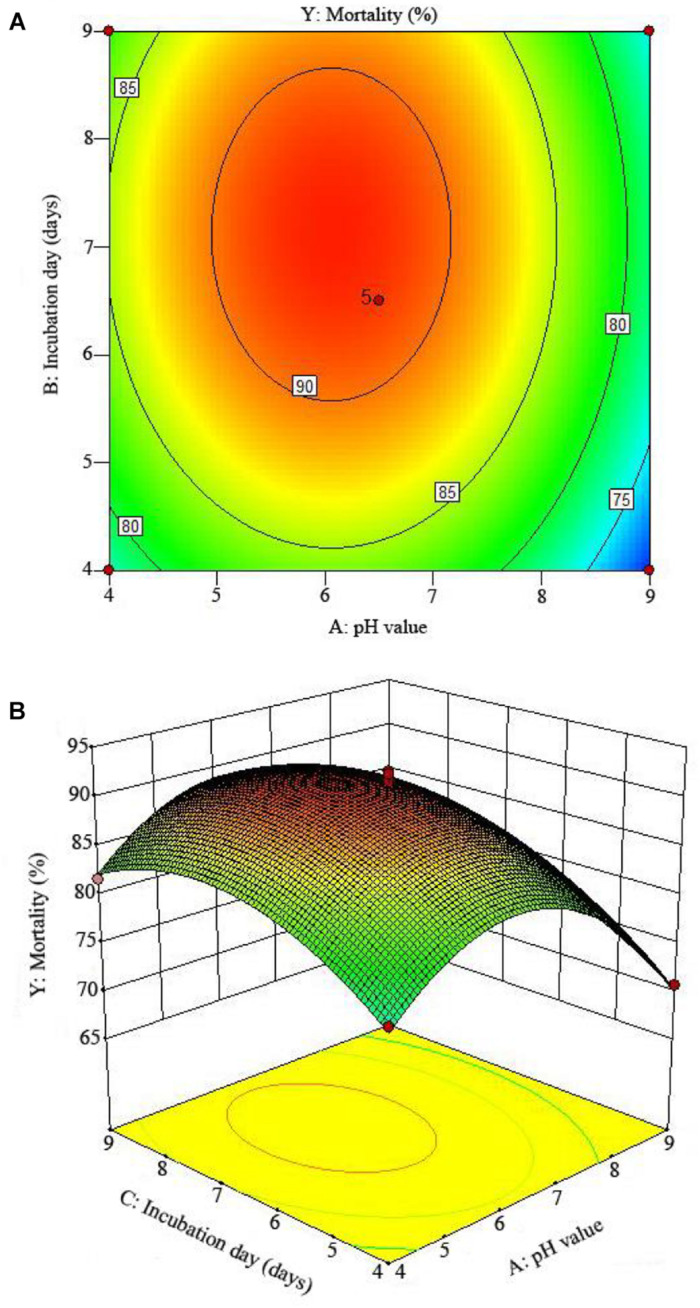
The mortality response to the interactive effects of pHs and incubation days on the nematicidal activity of *Trichoderma longibrachiatum* T6 against *H. avenae* as presented with **(A)** contour plots and **(B)** the 3D response surface presentation.

**FIGURE 4:**
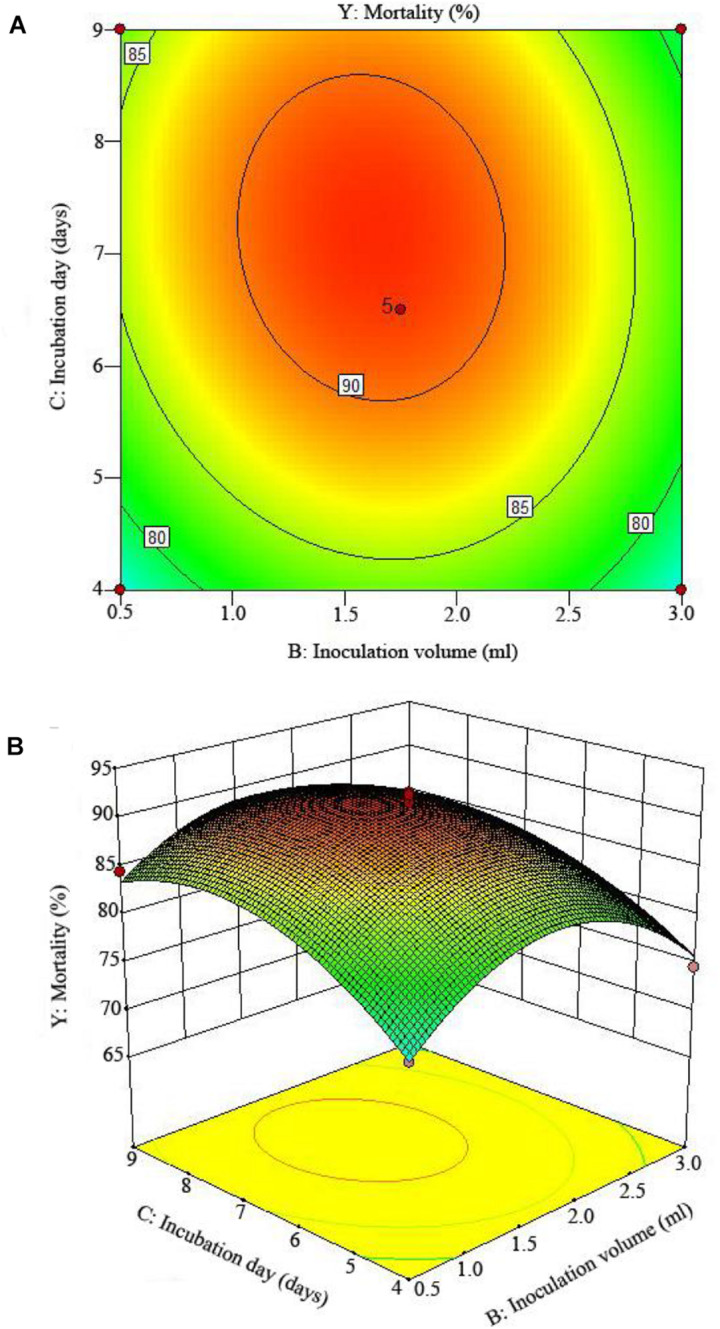
The mortality response to the interactive effects of inoculation volumes and incubation days on the nematicidal activity of *Trichoderma longibrachiatum* T6 against *H. avenae* as presented with **(A)** contour plots and **(B)** the 3D response surface presentation.

**FIGURE 5:**
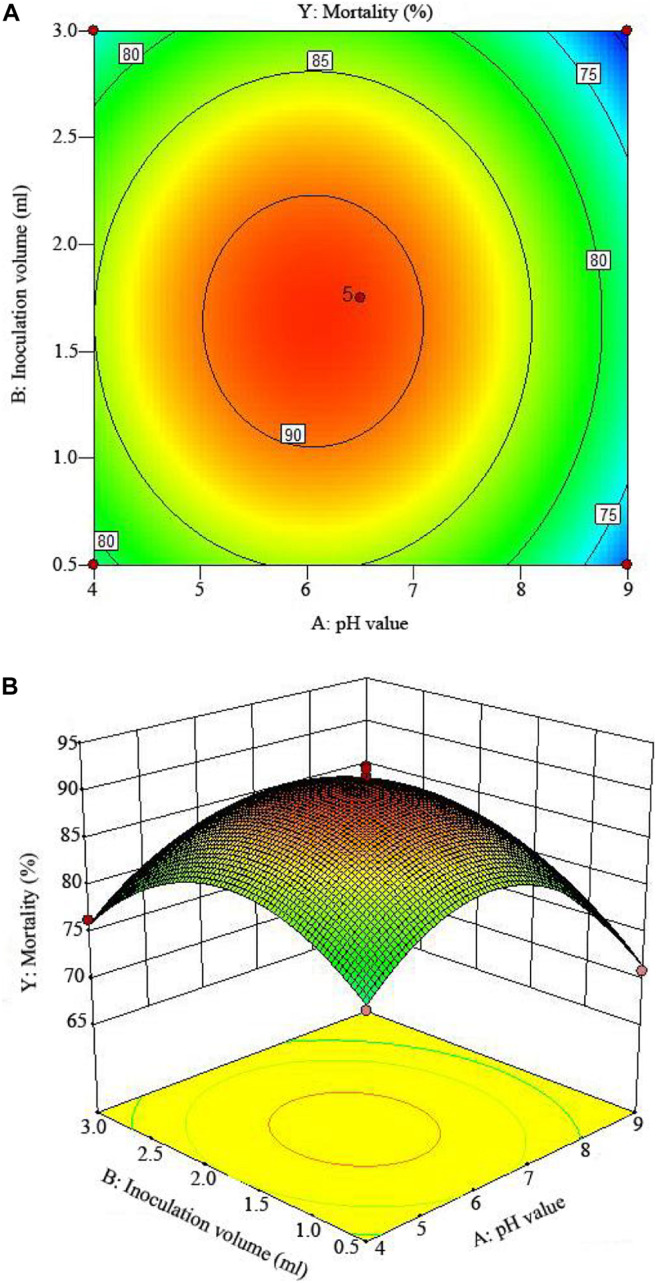
The mortality response to the interactive effects of pHs and inoculation volumes on the nematicidal activity of *Trichoderma longibrachiatum* T6 against *H. avenae* as presented with **(A)** contour plots and **(B)** the 3D response surface presentation.

The 2D contour plots and the 3D response surface plots further showed that the mortality was mainly determined by different combinations of two parameters, i.e., initial pH value and incubation day (Figures 3A,B), inoculation volume and incubation day (Figures 4A,B), and initial pH value and inoculation volume (Figures 5A,B). For the response contour plots (Figures 3A, 4A, 5A), the maximum mortality of 92.05% was obtained at initial pH, 6.06; inoculation volume, 1.62 ml; and incubation day, 7.15 days. Also, the 3D surface contour plots displayed the predicted mortality of 92.05% at the two-parameter combinations (Figures 3B, 4B, 5B). Moreover, these two-parameter combinations were found to have synergistic effects on the mortality in comparison to the data in Table 6.

### Verification of the Nematicidal Activity of T6 Culture Filtrates From Optimized Fermentation Media

The nematicidal activity (mortality) of the T6 culture filtrates from the optimized fermentation medium was determined again using the predicted optimal conditions as follows: initial pH, 6.06; inoculation volume, 1.62 ml; and incubation time, 7.15 days. The results indicated that the nematicidal activity of the T6 culture filtrates was significantly increased (Table 8). The mortality was 92.42% at 72 h after treatment, significantly higher than those of positive and negative controls (Table 8) and close to the predicted mortality of 92.05%. This demonstrated that the response quadratic polynomial model could adequately reflect the expected optimization.

**TABLE 8:** Verification of the nematicidal activity of *Trichoderma longibrachiatum* T6 culture filtrates against *H. avenae* at 24, 48, and 72 h after incubation under optimal conditions.

Treatments	Time (h)		
	24	48	72

	**Mortality (%)**		
T6 culture filtrates	48.34 ± 2.22 a	78.43 ± 2.53 a	92.42 ± 3.34 a
MM + Wheat bran-2	31.37 ± 2.54 b	68.58 ± 3.26 b	91.44 ± 3.32 b
Positive control	7.05 ± 1.38 c	13.45 ± 1.21 c	14.21 ± 1.37 c
Negative control	8.58 ± 0.89 c	10.07 ± 1.04 c	13.28 ± 1.29 c

*Data are means of 12 replicates ± standard error. Values in columns followed by different letters are significantly different at p < 0.05 based on Duncan’s new multiple range test using one-way ANOVA (n = 12). Negative control represents the J2s that were treated with sterilized water, whereas the positive control represents the J2s that were treated with the fermentation medium without T. longibrachiatum T6.*

In addition, the mortality of the T6 culture filtrates from the optimized fermentation medium was significantly different from that of the T6 culture filtrates from the MM + wheat bran-2 (G) medium. The nematicidal activity was increased approximately by as much as 1.07% compared with that before optimization (Table 8).

### Bio-control Efficacy in Greenhouse

The number of *H. avenae* J2s in soil was significantly decreased after the application of T6 fermentation and abamectin at 70 days after wheat seeds sowing in comparison to the two controls. Meanwhile, the treatments with T6 culture filtrates exhibited higher bio-control efficacy than the abamectin treatment in the greenhouse experiments. The bio-control efficacy of T6 culture filtrates was 83.88% at the 70^th^ day after wheat seeds sowing, whereas the bio-control efficacy of abamectin was 77.15% in comparison to wheat seedlings treated with sterilized water (Table 9).

**TABLE 9:** Verification of the bio-control activity of *Trichoderma longibrachiatum* T6 culture filtrates against *H. avenae* in greenhouse experiments at the 70th day after wheat seeds sowing.

Treatments	Number of juveniles in soil (per 200 g of soil)	Bio-control efficacy (%)
T6 culture filtrates	74.51 ± 3.24 c	83.88 ± 2.83 a
Abamectin	105.64 ± 6.75 b	77.15 ± 2.94 b
Positive control	452.83 ± 23.11 a	2.05 ± 0.21 c
Negative control	462.32 ± 25.44 a	_

*Data are means of 12 replicates ± standard error. Values in columns followed by different letters are significantly different at p < 0.05 based on Duncan’s new multiple range test using one-way ANOVA (n = 12). Negative control represents the wheat seedlings that were treated with sterilized water, whereas the positive control represents the seedlings that were treated with the liquid fermentation medium but not with T6 culture filtrates.*

## Discussion

In the present study, the MM + wheat bran-2 (wheat bran, 25 g; KH_2_PO_4_, 1 g; K_2_HPO_4_, 1.5 g; MgSO_4_⋅7H_2_O, 2 g; FeSO_4_⋅7H_2_O, 0.1 g) was identified as the most effective fermentation medium for the high nematicidal activity of the T6 culture filtrates among 10 fermentation media tested. To the best of our knowledge, this is the first report suggesting that MM + wheat bran-2 medium could increase the nematicidal activity of T6 culture filtrates against *H. avenae* J2s. This is consistent with the previous study of the fermentation medium and fermentation parameters in enhancing the bio-control ability of *T. viride* YC-108 mutant strain (Yan et al., 2012; Yang et al., 2016). Yan et al. (2012) reported that wheat bran, avicel, and soya-bean cake powder, nitrogen source, and soybean meal could promote and increase the cellulase production from the strain of *T. reesei* YC-108. In addition, Wang et al. (2014) revealed that the components of K_2_HPO_4_, beef extract, beef peptone, and glucose in a liquid fermentation medium improved the nematicidal activity of the nematode pathogenic strain *Xenorhabdus nematophila* HB301. It was found that the optimal enzymatic activity of the strain *T. harzianum* F-470 was obtained with the fermentation medium of crude state and at the cultural conditions of temperature 40°C and pH 5.5 (Wiater and Szczodrak, 2001). Sharma et al. (2014) found that the mortality of *M. incognita* J2s was increased to 100% if the nematode pathogenic strain *P. lilacinus* 6029 was fermented on the medium of Karanja cake than the Czapeck–Dox filtrate under the fermentation parameters of a lower pH and 15 days’ incubation time. It was also found that the fermentation parameters of incubation time, initial pH, and temperature were the most important factors for bio-control of root knot nematodes by *P. lilacinus* production (Yu et al., 2015).

Based on single-factor experiments and the Plackett–Burman design experiments, the optimal fermentation parameters of the MM + wheat bran-2 medium were an initial pH of 6, fermentation temperature at 28°C, rotating speeds at 180 rpm, fermentation media volume of 60 ml, inoculum volume of 1 ml, and incubation for 7 days. They made a significant effect on increasing the nematicidal activity of T6 culture filtrates against *H. avenae* J2s. Li et al. (2016) found, using the Plackett–Burman design, that cornmeal, glycerol, and initial pH were the most significant parameters for enhancing the production of chlamydospores of *T. harzianum* SH2303 in liquid fermentation. Through Plackett–Burman screening experiments, Yang et al. (2016) reported that three fermentation parameters such as initial pH, concentration of sucrose, and MgSO_4_⋅7H_2_O in a broth had significant effects on the nematicidal activity of *Simplicillium chinense* strain Snef5 against *M. incognita* J2s. By further study using the Box–Behnken design and RSM, Yang et al. (2016) redefined the optimal conditions for the strain *S. chinense* Snef5 fermentation as 6.74 initial pH, 4.19% sucrose, and 0.04% MgSO_4_⋅7H_2_O in the medium, and further enhanced the nematicidal activity of the strain Snef5. Similarly, in the current study, the optimal fermentation parameters for the nematicidal activity of T6 culture filtrates were redefined as initial pH, 6.06; inoculum quantity, 1.62 ml; and incubation time, 7.15 days. Under such optimized condition, the nematicidal activity and bio-control efficacy of T6 culture filtrates was significantly increased. The mortality results of the verification experiments under these conditions correlated well with the predicted mortalities with good Adj *R*^2^ and *R*^2^-values.

Our study demonstrated that RSM is a valuable tool to optimize the fermentation parameters (Trupkin et al., 2003; Li et al., 2007) due to its advantages of short test cycle, high accuracy, and low test frequency. The differences in the nematicidal activity between our study and previous studies may be due to different strains and their metabolites under different fermentation conditions. The outcome of our work provides a promising reference for future development of more effective and practical bio-nematicide fermentation processes (Ambat and Ayyanna, 2001). Despite the promising results achieved in this study, some issues like the active ingredient on the activity of T6 culture filtrates against *H. avenae* J2s need further investigation.

## Conclusion

The present study has highlighted the design, analysis, and optimization of the fermentation parameters of the beneficial fungus *T. longibrachiatum* T6 (T6) for its nematicidal activity and bio-control efficacy enhancement. To the best of our knowledge, the present work is the first report on the application of the Plackett–Burman design and RSM to screen and optimize the fermentation parameters on the bio-control potential of *T. longibrachiatum* against *H. avenae* in the surface and submerged fermentation techniques. Our results indicate that statistical analysis using the Plackett–Burman design and RSM is a valuable approach for studying and optimizing the combined effects of fermentation parameters of the beneficial fungi. The model obtained by the Plackett–Burman design and RSM analysis has better precision and reliability in maximizing the nematicidal activity of T6 culture filtrates compared with that before optimization. The model will help to provide a more effective and practical bio-nematicide fermentation process for the development of some novel commercial bio-control agents in the future.

## Data Availability Statement

All datasets presented in this study are included in the article/supplementary material.

## Author Contributions

SZ and YG conceived and designed the experiments with the help of BX. SZ performed most of the optimization of the fermentation media and parameter experiment and prepared the nematode samples. JZ, JL, and SZ analyzed the data, with the help of BX. SZ and JZ wrote the manuscript. SZ, YG, JZ, and BX revised and approved the final manuscript. All authors contributed to the article and approved the submitted version.

## Conflict of Interest

The authors declare that the research was conducted in the absence of any commercial or financial relationships that could be construed as a potential conflict of interest.

## Abbreviations

J2s: second stages juveniles
PDA: potato dextrose agar
RSM: response surface methodology
T6: *Trichoderma longibrachiatum* T6.
